# A short comment on statistical versus mathematical modelling

**DOI:** 10.1038/s41467-019-11865-8

**Published:** 2019-08-27

**Authors:** Andrea Saltelli

**Affiliations:** 1 Centre for the Study of the Sciences and the Humanities (SVT), University of Bergen (UIB), 5020 Bergen, Norway; 2 Open Evidence Research, Universitat Oberta de Catalunya (UOC), 08018 Barcelona, Spain

**Keywords:** Environmental sciences, Mathematics and computing, Computational science

## Abstract

While the crisis of statistics has made it to the headlines, that of mathematical modelling hasn’t. Something can be learned comparing the two, and looking at other instances of production of numbers.Sociology of quantification and post-normal science can help.

While statistical and mathematical modelling share important features, they don’t seem to share the same sense of crisis. Statisticians appear mired in an academic and mediatic debate where even the concept of significance appears challenged, while more sedate tones prevail in the various communities of mathematical modelling. This is perhaps because, unlike statistics, mathematical modelling is not a discipline. It cannot discuss possible fixes in disciplinary fora under the supervision of recognised leaders. It cannot issue authoritative statements of concern from relevant institutions such as e.g., the American Statistical Association or the columns of Nature.

Additionally the practice of modelling is spread among different fields, each characterised by its own quality assurance procedures (see^1^ for references and discussion). Finally, being the coalface of research, statistics is often blamed for the larger reproducibility crisis affecting scientific production^2^.

Yet if statistics is coming to terms with methodological abuse and wicked incentives, it appears legitimate to ask if something of the sort might be happening in the multiverse of mathematical modelling. A recent work in this journal reviews common critiques of modelling practices, and suggests—for model validation, to complement a data-driven with a participatory-based approach, thus tackling the dichotomy of model representativeness—model usefulness^3^. We offer here a commentary which takes statistics as a point of departure and comparison.

For a start, modelling is less amenable than statistics to structured remedies. A statistical experiment in medicine or psychology can be pre-registered, to prevent changing the hypothesis after the results are known. The preregistration of a modelling exercise before the model is coded is unheard of, although without assessing model purpose one cannot judge its quality. For this reason, while a rhetorical or ritual use of methods is lamented in statistics^2^, it is perhaps even more frequent in modelling^1^. What is meant here by ritual is the going through the motions of a scientific process of quantification while in fact producing vacuous numbers^1^.

All model-knowing is conditional on assumptions^4^. Techniques for model sensitivity and uncertainty quantification can answer the question of what inference is conditional on what assumption, helping users to understand the true worth of a model. This understanding is identified in ref. ^3^ as a key ingredient of validation. Unfortunately, most modelling studies don’t bother with a sensitivity analysis—or perform a poor one^5^. A possible reason is that a proper appreciation of uncertainty may locate an output on the right side of Fig. 1, which is a reminder of the important trade-off between model complexity and model error. Equivalent formulations of Fig. 1 can be seen in many fields of modelling and data analysis, and if the recommendations of the present comment should be limited to one, it would be that a poster of Fig. 1 hangs in every office where modelling takes place.

**Fig. 1:**
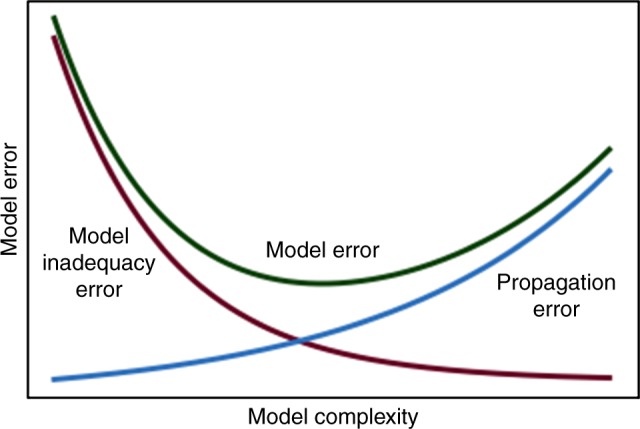
Model error as ideally resulting from the superposition of two curves: (i) model inadequacy error, due to using too simple a model for the problem at hand. This term goes down by making the model more complex; (ii) error propagation, which results from the uncertainty in the input variables propagating to the model output. This term grows with model complexity. Whenever the system being modelled in not elementary, overlooking important processes leaves us on the left-hand side of the plot, while modelling hubris can take us to the right-hand side

In modelling—as is the case of statistics, one can expect a mix of technical and normative problems—the latter referring to expectations, interests, values and policies being touched by the modelling activity. In cost-benefit analyses an estimate of return giving a range from a large loss to a large gain may not be what the client wishes to hear. The analysts may be tempted to “adjust” the uncertainty in the input until the output range is narrower and conveniently located in friendlier territory. Integrated climate-economy models pretend to show the fate of the planet and its economy several decades ahead, while uncertainty is so wide as to render any expectations for the future meaningless. In economics, models universally known to be wrong continue to play a role in economic policy decisions, while the neologism ‘mathiness’ has been proposed for the use of mathematics in models to veil ideological stances. Disingenuous pricing of opaque financial products is held as partly responsible for the onset of the last recession: modellers chose to calibrate the pricing of bundles of mortgages based on data for the real estate market in an up-swing period. Needless to say, these calibrations conveniently ignored what would happen when the market took a turn for the worse. Transport policy offer a curious example where a model requires as an input how many people will be sitting in a car on average decades from now. See ref. ^1^ for the references to the cases just described. More examples are described in ref. ^6^, portraying flawed models used to justify unwise policies in evaluation of fisheries’ stock, AIDS epidemics, mill tailing, coastal erosion, and so on. Among those, studies for the safety of an underground disposal of radioactive waste stand out for providing what the authors in^6^ call “A million years of certainty”, achieved thanks to a huge mathematical model including 286 sub-models.

Modelling hubris may lead to “trans-science”, a practice which lends itself to the language and formalism of science but where science cannot provide answers^7^. Models may be used as a convenient tool of displacement – from what happens in reality to what happens in the model^8^. The merging of algorithms with big data blurs many existing distinctions among different instances of quantification, leading to the question “what qualities are specific to rankings, or indicators, or models, or algorithms?”^9^ Thus the problems just highlighted are likely to apply to all of these instances, as shown by the recent alarm about unethical use of algorithms^10^, the disruptive use of artificial intelligence exemplified by Facebook, or the well documented problems with the abuse of metrics^11^, which is now reflected in an increasing militancy against statistical and metrical abuses^12^.

This is not an indictment of mathematical modelling. Modelling is essential to the scientific enterprise. When Steven Shapin, a scholar studying science and technology, talks about “invisible science”—meaning scientific and technological products which improve our life—one chapter could be devoted to “invisible models” underpinning these technologies. The malpractices alluded to above are all different: not only a racist algorithm is different from an audacious cost-benefit analysis, or a low-powered statistical study. Even within modelling, different problems are at play. Modelling hubris has its counterpart in living in an idealised model-land of appealing simplicity but scarce realism^6^.

Hence, recipes cannot be prescriptive or universal. The following could help (see ref. ^1^ for details):

- Memento Fig. 1.
- Mathematical modelling could benefit from structure and standards based on statistical principles including a systemic appraisal of model uncertainties and parametric sensitivities.
- Statistics could help by internalising these into its own syllabi and practices.
- Models–including algorithms, should be made inherently interpretable.
- For key models used in policy, peer review should be extended to include auditing by an extended community involving a plurality of disciplines and interested actors, leading to model pedigrees, as discussed on this journal^3^ and more diffusely in ref. ^1^.
- Audits could be used to uncover a model’s underlying, unspoken, metaphors^1^.

To put the prescriptions into practice a movement of resistance is needed, perhaps along the lines of the so-called statistical activism^12^. This kind of resistance is familiar to scholars gathered around post-normal science (PNS)^13^. The foundational works^14,15^ of PNS’ fathers Silvio Funtowicz and Jerome R. Ravetz see model quality in terms of fitness for purpose. As noted in ref. ^3^ this view—with would entail reconsidering the model any time to see whether the purpose or the question put to the model are changed—is still a minority view in the modelling community. PNS suggests an approach to the use of models which is more reflexive—i.e., the analyst is part of the analysis, and participatory—including an extended peer community. While this vision is gaining new traction^3^ more could be done. A new ethics of quantification (https://www.uib.no/en/svt/127044/ethics-quantification) must be nurtured, which takes inspiration from a long tradition of sociology of numbers; Pierre Bourdieu^12^ and Theodor Porter^16^ come to mind. What the authors in ref. ^3^ chose to call the distinction between a positivistic and a relativistic philosophy in model validation needs to be overcome for progress to be achieved.
